# Will Climate Change, Genetic and Demographic Variation or Rat Predation Pose the Greatest Risk for Persistence of an Altitudinally Distributed Island Endemic?

**DOI:** 10.3390/biology1030736

**Published:** 2012-11-23

**Authors:** Catherine Laura Simmons, Tony D. Auld, Ian Hutton, William J. Baker, Alison Shapcott

**Affiliations:** 1 Faculty of Science Health, Education and Engineering, University of the Sunshine Coast, Maroochydore DC, QLD 4558, Australia; Email: Csimmons@usc.edu.au; 2 Office of Environmentand Heritage (NSW), P.O. Box 1967 Hurstville, NSW 2220, Australia; Email: tony.auld@environment.nsw.gov.au; 3 P.O. Box 157, Lord Howe Island, NSW 2898, Australia; Email: ianhutton@clearview.com.au; 4 Royal Botanic Gardens, Kew, Richmond, Surrey, TW9 3AB, UK; Email: w.baker@kew.org

**Keywords:** climate change, genetic variation, growth rates, population growth

## Abstract

Species endemic to mountains on oceanic islands are subject to a number of existing threats (in particular, invasive species) along with the impacts of a rapidly changing climate. The Lord Howe Island endemic palm *Hedyscepe canterburyana* is restricted to two mountains above 300 m altitude. Predation by the introduced Black Rat (*Rattus rattus*) is known to significantly reduce seedling recruitment. We examined the variation in *Hedyscepe* in terms of genetic variation, morphology, reproductive output and demographic structure, across an altitudinal gradient. We used demographic data to model population persistence under climate change predictions of upward range contraction incorporating long-term climatic records for Lord Howe Island. We also accounted for alternative levels of rat predation into the model to reflect management options for control. We found that Lord Howe Island is getting warmer and drier and quantified the degree of temperature change with altitude (0.9 °C per 100 m). For *H. canterburyana*, differences in development rates, population structure, reproductive output and population growth rate were identified between altitudes. In contrast, genetic variation was high and did not vary with altitude. There is no evidence of an upward range contraction as was predicted and recruitment was greatest at lower altitudes. Our models predicted slow population decline in the species and that the highest altitude populations are under greatest threat of extinction. Removal of rat predation would significantly enhance future persistence of this species.

## 1. Introduction

Oceanic island floras are of high conservation value as they are isolated, geographically diverse and support endemic plant species that have evolved *in situ* and distinctive assemblages [1,2]. More than half of all palm species are endemic to islands, with the Pacific Ocean particularly high in palm genus endemism [3,4]. There are four palm species in three endemic genera on Lord Howe Island (LHI) in the western Pacific Ocean, (tribe Areceae, Arecoideae), which are thought to have arrived in three colonisation events [5,6].

Islands of volcanic origin, such as LHI, can feature steep mountain gradients, causing adiabatic cooling and orographic cloud formation at higher elevations [7]. Under such clouds, microclimates with reduced radiation and daily temperature range, combined with increased moisture can occur [7,8]. Cloud forest ecosystems can form in such conditions and component species may be adapted to conditions within a specific elevational range. For example, gradations in physiology, morphology and development may occur in response to the environmental conditions, due to different gene frequencies or phenotypic plasticity that have developed over time [9,10,11].

**Figure 1:**
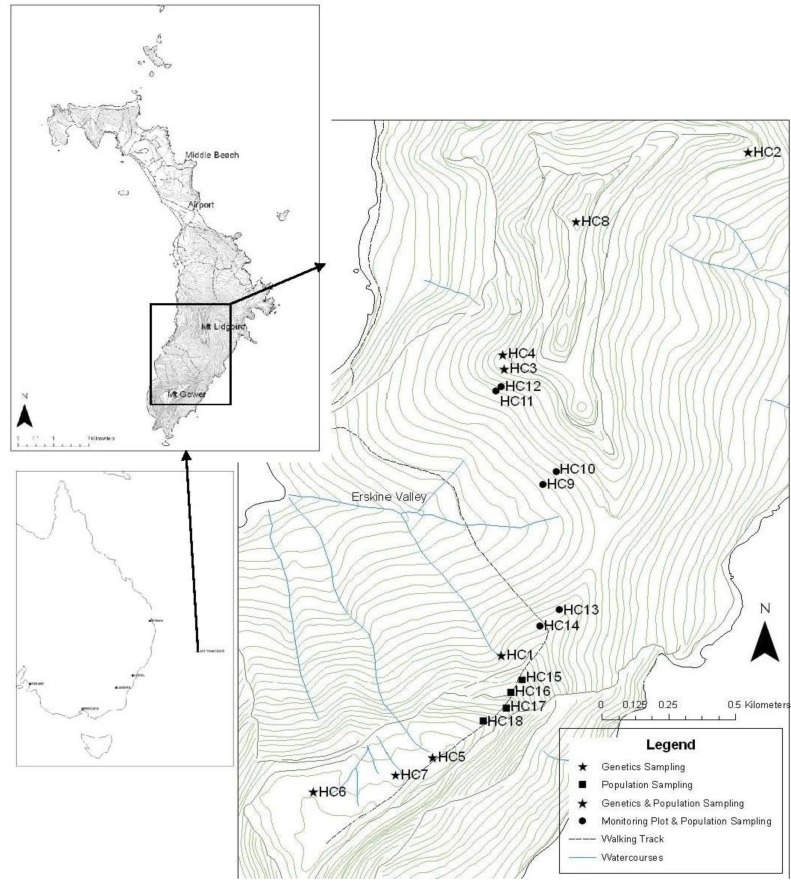
Map showing the location of Lord Howe Island in relation to Australia, highlighting the southern mountains, Mt Lidgbird (777 m) and Mt Gower (875 m). Enlargement map showing sites where *H. canterburyana* was sampled: genetics sampling sites, population demographic sampling sites, long term monitoring sites and combination sampling sites. The steep topography is indicated with 20 m contour lines from sea level to 860 m.

Lord Howe Island is a small (1,455 Ha) volcanic landmass in the South Pacific Ocean approximately 570 km from Australia (Figure 1) [12]. The island has a unique floristic assemblage, with 44% of plant species endemic to the island [12]. Extensive orographic cloud forms over two mountains that dominate the island, Mt Gower (875 m) and Mt Lidgbird (777 m) [13]. Mossy cloud forest, which alone supports 34% of the island’s endemic plant species including two palms [14], occupies less than 0.5 km^2^ on LHI including the summit of Mt Gower. One of these palms, *Hedyscepe canterburyana* (C. Moore & F. Muell) H. Wendl & Drude, is listed as “vulnerable” on the IUCN Red List and occurs between 300–850 m altitude into the cloud forest on Mt Lidgbird and Mt Gower as well as their upper slopes [3,15]. *Hedyscepe canterburyana* is a monoecious palm with insect-pollinated protandrous inflorescences and large red fruits (~3 × 4.4 cm) that are potentially dispersed by the Lord Howe Pied Currawong (*Strepera graculina crissalis*) [3].

Species with small or isolated geographic ranges and narrow environmental tolerances are most at risk under climate change [16,17]. In response to climatic warming, species at particular altitudes are expected to migrate upwards [18,19,20]. Such range shifts may be characterised by reduced population growth at lower altitudes and have already been reported in the northern hemisphere [21,22,23]. The four LHI endemic palm species are distributed along an elevation gradient [3]. *Howea forsteriana* occurs on calcareous and basaltic soils at lower altitudes (to 350 m), *Howea belmoreana* occurs on basaltic soils only at lower altitudes (to 400 m) and *Lepidorrhachis mooreana* is restricted to the mountain summits (750–875 m). The topic of this study, *Hedyscepe canterburyana*, co-occurs with *Howea belmoreana* and *Lepidorrachis mooreana* [3,24]. Climate change may alter the pattern of cloud formation or cloud height over montane habitats [7,25,26] and is thought to be the most critical threat to the survival of endemic flora on Lord Howe Island due to its limited scope for migration [27,28]. Demographic structure and population dynamic modelling studies are easily undertaken on palms and have potential to be used for gauging climate effects on populations [29,30,31].

Where geography limits the possibility of adaptive migration, survival of a species may depend on their ability to adjust to the changed climatic conditions *in situ* [32]. Isolated species with low genetic variation are at high risk [33,34]. High levels of genetic diversity and strong directional selection pressures facilitate adaptation [34,35]. However, selection acts over generations, and for long lived species this may be slower than the rate of current climatic changes [36,37].

The possession of phenotypic plasticity may buffer individuals against the short term effects of climate change [32,36,38,39]. Morphology has been found to vary across the distribution of several palm species suggesting some plasticity in this group of plants [40,41]. Timing of plant flowering is known to vary across elevation gradients, due to adiabatic temperature changes and temperature-specific flowering initiation [42]. Where flowering is asynchronous, restricted gene flow has resulted in genetic structuring [43,44]. Phenological changes have been reported in some species in response to recent climatic changes [45,46] which may affect long term gene exchange [42,47].

In addition to the potential impacts of climate change, many island plants are already vulnerable to predation by invasive species [48,49]. Rats such as Black Rats (*Rattus rattus*) cause “recruitment depression” for many plants on islands and may be the cause of extinction of some species [50,51,52,53]. Island endemic palms may be affected by synergies between climate change and seed predation by rats. Black rats arrived on LHI in 1918 and quickly became widespread and abundant. Rat control measures are currently undertaken on a small part of the island [54]. Consumption of fruit by invasive black rats has been documented to severely reduce the levels of recruitment of *H. canterburyana* seedlings in unbaited areas [24].

Conservation actions for the next several decades urgently require guidance based on a defendable scientific understanding of the potential impacts posed by a suite of threats to plant communities [55,56]. This study aims to test likely impacts of climate change on a highly restricted plant species occurring over an elevation gradient. Specifically we examined if genetic variation, morphology, reproductive output and demographic structure for the Lord Howe Island endemic palm *H. canterburyana* varied across an altitude gradient and what impacts this may have on its long-term viability and persistence. In addition, factors such as aspect, protection in shade and site differences were also investigated for potential association with genetic composition and demographic structure. To inform predictions of climate changes we quantified simple elevation lapse rates (adiabatic temperature change) and examined evidence of climatic changes on LHI in the last century using historical climatic records. Population models were used to compare the risk of extinction at different elevations as an indicator of potential upward movement in the species distribution due to climatic changes. This incorporated the impact of possible rat control measures.

## 2. Results and Discussion

### 2.1. Climatic Conditions

**Figure 2:**
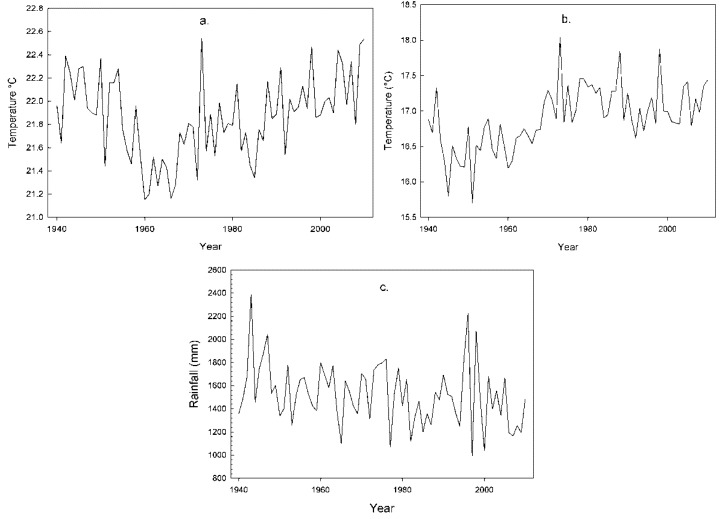
Climate on Lord Howe Island, 1940–2010. (**a**) Mean annual maximum temperatures. (**b**) Mean annual minimum temperatures. (**c**) Total annual rainfall.

Lord Howe Island has experienced a variable annual climate in the last 70 years, with the 2000–2009 decade experiencing the lowest mean annual rainfall (1,362 mm; Figure 2), in line with droughts experienced during the same time-frame across the east of Australia. Annual minimum and maximum temperatures significantly increased across decades (*p* < 0.01, *r_s_* = 0.082; *r_s_* = 0.026; Table 1; Figure 2). Mean minimum temperatures increased by 0.6 °C in total since 1940 with the period of greatest increase occurring between the 1960s and 1970s. Maximum temperatures increased by 0.025 °C on average each decade (*r^2^* = 0.02). Annual rainfall was negatively correlated with time (*p* < 0.01, *f* = 2.647, *r_s_* = −0.281). The rates of change found for LHI in this study concur with worldwide climatic changes since the mid-twentieth century reported on a regional scale [20,57,58]. The annual variability in the climate data reported here are common in Australia and New Zealand, partially due to El Niño influences [59].

As expected, temperature varied significantly between altitudes, decreasing significantly with increasing altitude (*p* < 0.01; mean *r_s_* = −0.546; min. *r_s_* = −0.567; max. *r_s_* = −0.457). The adiabatic lapse rate for Mt Gower was estimated to be −0.9 °C per 100 m increase in altitude (*t* = 38.514, *p* < 0.01, *r^2^* = 0.383). This is greater than adiabatic temperature lapse rates reported for Hawaii, Borneo and Madagascar which range from 0.4 to 0.6 °C decrease per 100 m increase in elevation [20,60,61]. Our figures are consistent with estimations by the Lord Howe Island Board [13] and those reported for the Krakatau Islands [62]. Simulations predict cloud formation lifting to higher altitudes and for cloud forest areas to experience reduced water availability, increased temperature and daily temperature variation [7,14,26,63]. Based on the current temperatures at low elevations and climatic changes already experienced on LHI, the future warm temperature limit for *H. canterburyana* is predicted to rise to elevations of 426–500 m by 2030 and 451–710 m by 2070. Thus an upslope range shift in the last half century may already be occurring for *H. canterburyana* and would be shown by current recruitment failure or increased mortality at lower elevations. 

**Table 1:** Summary of statistical results 1940–2009 mean monthly minimum (*Min*.) and maximum (*Max*.) temperatures; results of Spearman’s rank correlation for time (*r_s_*); ANOVA (*f*) testing for difference in decades for each month; statistical values * *p* < 0.05, ** *p* <0.01, *** *p* < 0.001.

	*Mean Max. Temperature*		*Mean Min. Temperature*	
	decade (*r_s_*)	decades (*f*)	decade (*r_s_*)	decade (*f*)
*January*	−0.050 **	16.525 **	0.199 **	23.580 **
*February*	−0.005	18.309 **	0.212 **	20.365 **
*March*	−0.063 **	18.37 **	0.131 **	15.877 **
*April*	0.029	6.683 **	0.106 **	14.894 **
*May*	0.134 **	11.679 **	0.151 **	21.133 **
*June*	0.181 **	20.095 **	0.103 **	4.054 **
*July*	0.153 **	21.768 **	0.119 **	8.096 **
*August*	0.124 **	14.156 **	0.113 **	8.596 **
*September*	0.172 **	24.201 **	0.168 **	13.155 **
*October*	0.052 **	6.541 **	0.122 **	8.385 **
*November*	−0.074 **	17.621 **	0.111 **	7.679 **
*December*	−0.041	29.192 **	0.158 **	16.540 **
*Annual*	0.026 **	30.058 **	0.082 **	41.473 **

### 2.2. Population Demography

The mean density (*D*) of *H. canterburyana* individuals was 1,644 plants/Ha, with an average distance to nearest neighbour of 1.2 m (*NND1*; Table 2). Density (*D*) of trunked *H. canterburyana* increased with increasing elevation (*D r_s_* = 0.655; *NND1 r_s_* = −0.675; *p* < 0.05; Table 2). Individuals were more sparsely distributed in sites shaded by Mt Lidgbird (Table 2).

**Figure 3:**
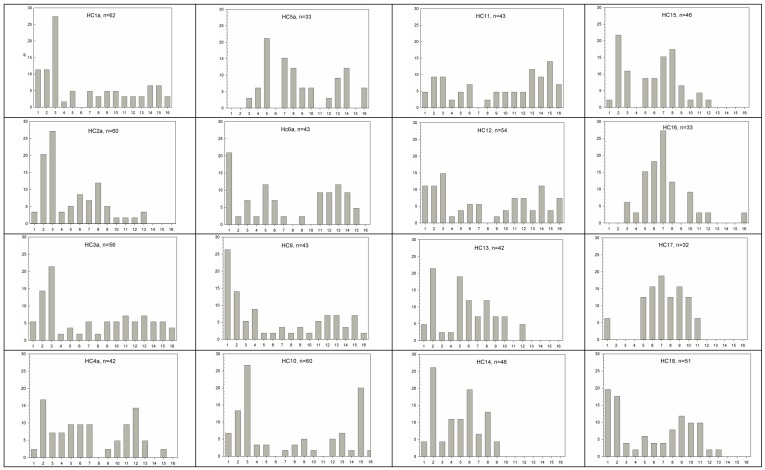
Proportion of *H. canterburyana* individuals in each height class calculated at each site (%; *y axis*). Height classes (*x axis*) 1 = no trunk >0.6 m, 2 = no trunk 0.6–1.2 m, 3 = no trunk >1.2 m, 4 = trunk <0.5 m, 5 = trunk 0.5–1 m, 6 = trunk 1–1.5 m, 7 = trunk 1.5–2 m, 8 = trunk 2–2.5 m, 9 = trunk 2.5–3 m, 10 = trunk 3–3.5 m, 11 = trunk 3.5–4 m, 12 = trunk 4–4.5 m, 13 = trunk 4.5–5 m, 14 = trunk 5–6 m, 15 = trunk >6 m.

Population structure varied between sites, but generally there were greater numbers of adults than juveniles with some apparent historical pulses of recruitment (Figure 3). The presence and abundance of a non-trunked palm “juvenile bank” is a key measure of regeneration to maintain population size [6,24,64]. On average, one new seedling results from every three reproductively active individuals (0.33 seedlings per *RI*Table 3). Much greater recruitment and a larger juvenile bank is reported for wild populations of close relative *Rhopalostylis sapida* [29]. *Ex-situ* germination of *H. canterburyana* is reported to have a 70% success rate [65], while in the wild, the juvenile bank has been shown to be greatly enhanced in sites that are baited to control rats compared to unbaited sites [24]. Thus, it could be rationalised that the low recruitment levels are due in part to invasive black rats.

We expected that there would be greater seedling recruitment at higher altitudes due to moister conditions. In contrast to expectations, regeneration was greater at low altitudes in *H*. *canterburyana* (*r_s_*= −0.542, *t* = 3.278, * p* < 0.05). There was a significant positive correlation between altitude and “Sub-Adults” (*r_s_* = 0.629). Altitude was negatively correlated with the abundance of tallest trunked individuals (>6 m tall; *p* < 0.01, *r_s_* = −0.642).

**Table 2:** Summary of *H. canterburyana* population density and growth characteristics, Mt Gower and Mt Lidgbird means and statistical results for factors that may contribute to variation. Number of individuals surveyed (*n*) =760. Density of the site (*D*), average distance to one and three nearest neighbours (*NND1*, *NND3*), average height (*H_1_*), number of leaf scars per metre (*R/m*), diameter at breast height (*DBH*). Standard errors in parenthesis Results of Spearman’s rank correlation for altitude and aspect (*r_s_*); results of Independent T-tests (*t*) for the effects of mountain, exposure and altitude; ANOVA (*f*) between populations at different cardinal aspects. For all tests * *p* < 0.05, ** *p* < 0.01, *** *p* < 0.001.

**	*Total/mean*	*Mt Gower*	*Mt Lidgbird*	*Altitude (rs)*	*Aspect (rs)*	*Mountain (t)*	*Exposure (t)*	*Aspect (f)*	*Altitude (f)*
Non trunked & trunked									
*D (ha)*	1,643.6 (0.07)	2,003 (203)	1,182 (173)	0.418	−0.282	2.966 **	1.721	2.233	−2.214 *
*NND1 (m)*	1.2 (0.07)	1.1 (0.07)	1.3 (0.107)	−0.338	0.338	−2.055	−1.532	1.626	1.343
*NND3 (m)*	2.5 (0.15)	2.1 (0.10)	2.9 (0.20)	−0.588 *	0.373	−4.428 ***	−2.767 *	1.927	2.834 *
Trunked									
*D (ha)*	1,102.3 (129.59)	1,423.3 (138.9)	689.6 (111.5)	0.655 **	−0.417	3.940 **	2.410 *	4.749 *	−4.212 **
*NND1 (m)*	1.7 (0.132)	2.7 (0.17)	4.5 (0.52)	−0.674 **	0.513 *	−2.683 **	−1.111	3.086	2.357 *
*NND3 (m)*	3.5 (0.33)	1.4 (0.08)	1.9 (0.23)	0.511 *	−0.818 **	−3.750 **	−1.968	2.018	3.013 **
**									
*H_1_*	2.9 (0.08)	2.4 (0.094)	3.5 (0.149)	−0.220 **	0.239 **	−6.466 ***	−8.868 ***	4.944 ***	4.055 ***
*R/m*	43.0 (1.06)	43.5 (1.52)	42.4 (1.43)	−0.078	−0.046	0.539	0.415	5.495 ***	−2.454 *
*DBH*	9.2 (0.24)	8.4 (0.30)	10.1 (0.36)	−0.252 **	0.219 **	−3.570 ***	−3.935 ***	14.553 ***	2.269 *

**Table 3:** Summary of reproductive measures of *H. canterbuyana RI* (%), the proportion of population at reproductive age with reproductive activity; Mean *nS/RI* the mean number of seedlings per actively reproductive individual; *Inf*/RI inflourescence per reproductive individual; *MRO*, maximum reproductive output, the sum of all fruit abundance; Mean *MRO*, average maximum reproductive output per reproductive individual; *mMRO*, mean maximum reproductive output of mature sized fruits; *iMRO*, mean maximum reproductive output of immature sized fruits. Standard errors in parenthesis. Results of Spearman’s rank correlation for altitude and aspect (*r_s_*); results of Independent T-tests (*t*) for the effects of mountain, mountain shade and high and low altitudes; ANOVA (*f*) between populations at different cardinal aspects. * *p* < 0.05, ** *p* < 0.01, *** *p* < 0.001.

**	*Total*	*Mt Gower*	*Mt Lidgbird*	*Altitude (rs)*	*Altitude (t)*	*Aspect (rs)*	*Mountain (t)*	*Exposure (t)*	*Aspect (f)*
*RI (%)*	44.3	41.52 (7.54)	47.87 (5.77)	0.165	−1.971	0.357	0.704	−0.14	2.229
*nS/RI*	0.15 (0.40)	0.13 (0.045)	0.17 (0.074)	−0.349	1.655	−0.574 *	0.489	−0.037	2.564
*Inf/RI*	2.07 (0.08)	2.05 (0.31)	1.78 (0.16)	0.165	−1.971	0.357	0.704	−0.140	2.229
*MRO*	13.130	452.2 (255.61)	608.6 (150.20)	0.295 **	−3.499 **	−0.221 **	2.780 **	2.748 **	5.342 ***
*mMRO*	79.3 (6.79)	96.9 (13.79)	67.6 (6.29)	0.187	−3.255 **	−0.311	2.149 *	2.184 *	0.729
**									
*Developing Splade*	0.48	5.97 (5.52)	0	0.112	0.668	−0.240	0.946	0.714	0.322
*Half-Sized Green Fruit*	28.47	29.06 (7.62)	11.40 (4.02)	0.643 *	−5.637 ***	0.083	1.882	0.708	4.401 *
*Full-Sized Green Fruit*	39.23	28.97 (7.03)	48.36 (7.66)	−0.416	2.563 *	0.095	−1.856	−2.359 *	2.243
*Full-Sized Mottled Fruit*	4.07	1.12 (0.57)	5.30 (2.28)	−0.165	0.854	0.435	−1.995	−3.512 **	2.211
*Full-Sized Red Fruit*	0.48	0.21 (0.21)	0.36 (0.36)	0.141	−0.159	0.094	−0.375	−0.794	0.971
*Old*	27.03	34.66 (8.13)	34.05 (10.29)	0.088	0.12	−0.024	0.047	1.546	1.845

There was a weak yet significant correlation (*r_s_* = 0.380, *p* < 0.001) between the population structure and environmental attributes. A significantly greater proportion of “Adult” trunked individuals 4–4.5 m in height were found in sites with a northwest aspect (*p* < 0.05, *f* = 5.480) and the proportion of “mature adults” was significantly greater (Table 3) in sites in the protection of Mt Lidgbird. Low altitude sites, in the protection of Mt Lidgbird, with a south-east or east aspect displayed most continuous recruitment and individuals here had greater *DBH* and inferred longer plant ages (Table 2). Svenning [66] notes that a few hundred metres of change in altitude affects palm distribution in Ecuador, possibly due to frequent cloud formation at high altitudes. The lower altitude slopes of Mt Gower and Mt Lidgbird are steep (>29°) and composed of rock-fall debris which may aid drainage. Well drained forests have been known to increase prevalence of palm seedlings and juveniles in some species [66,67]. The potential role of differential seed predation by rats at different altitudes is unknown. 

We expected plants to respond to temperature change by growing more slowly at higher altitudes, have a greater *DBH*, and shorter trunk heights, as reported for other plant species [11,68]. Consistent with this hypothesis, trunked heights for *H. canterburyana* were weakly but significantly correlated with altitude (Table 2). However, individuals at high altitude sites had significantly smaller *DBH* (Table 2). Growth rate, measured by the number of rings per metre (*R*/*m*), averaged 43 rings per metre, but was highly variable within and among sites. In contrast to expectations, growth rate was significantly greater (fewer rings per metre) in individuals at high altitude sites compared to low altitude sites (Table 2). *Hedyscepe canterburyana* individuals at higher altitudes subject to increased cloud cover were also found at higher densities, indicating individual adults survive well in this environment. The palm may be more responsive to moisture in a micro-climate than to temperature, or alternatively, the lower altitude sites may be near the upper temperature tolerance of the species. Temperature is the main factor controlling palm distribution worldwide, but moisture also influences palm distribution and growth [3,69].

### 2.3. Fecundity and Phenology

**Figure 4:**
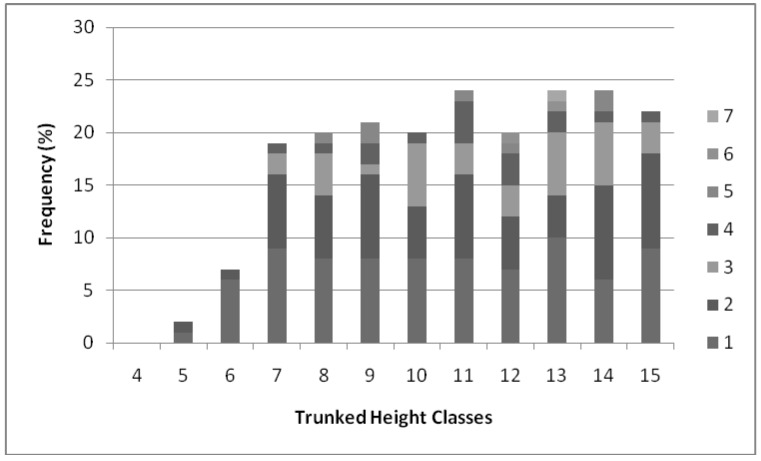
The percentage of *H. canterburyana* in each height class with each number of inflorescences present across all sites combined. Colours indicate the number of inflorescences, see legend. Height classes, 4 = trunk <0.5 m, 5 = trunk 0.5–1 m, 6 = trunk 1–1.5 m, 7 = trunk 1.5–2 m, 8 = trunk 2–2.5 m, 9 = trunk 2.5–3 m, 10 = trunk 3–3.5 m, 11 = trunk 3.5–4 m, 12 = trunk 4–4.5 m, 13 = trunk 4.5–5 m, 14 = trunk 5–6 m, 15 = trunk > 6 m.

The abundance of fruit produced, percentage of the population flowering, and the number of infructescences produced per individual, are variable amongst palms [68,70,71]. An average of two inflorescences were produced per reproductively active *H. canterburyana* individual (*Inf*). There were no significant correlations (*p* > 0.05) between inflorescence and population structure or environmental matrices. The number of inflorescences produced on each plant was correlated with the trunk height (*H_1_*; * r_s_* = 0.509; Table 2). *Hedyscepe canterburyana* individuals begin producing inflorescences once their trunk reaches 0.5 m, multiple inflorescence production commences above 1.5 m, peaks at 4.01–5 m (when individuals produce up to seven inflorescences) and declines at 5 m (Figure 4). The number of infructescences produced per individual in a year for *H. canterburyana* was comparable to that of close relative *Rhopalostylis sapida* [29]. *Hedyscepe canterburyana* has a comparable, though slightly greater proportion of individuals with reproductive structures present than the other Lord Howe Island palms [6,72]. Reproduction was variable between years with 24% of the *H. canterburyana* individuals (*RI*) having reproductive structures in 2008, compared with 44% in 2010. This may relate to variation in climatic conditions between those years. The effective population size for *H. canterburyana*, based on reproductive variability, is estimated to be approximately 50% of the census population, lower than that based on genetic estimates (*Ne* = 74%).

In *H. canterburyana*, higher elevation sites had significantly (*p* < 0.01) more mature sized fruits *(mMRO)* and greater overall reproductive output (*MRO*, *mMRO*; Table 3) compared to lower altitude sites. The abundance of unripe, half sized green fruit significantly increased with altitude (Table 3). Fruit produced, percentage of population flowering and the number of infructescences are measures of reproductive success, affecting recruitment [68,70,71]. Overall we found that reproductive output was greater at higher altitudes. Given that seedling abundance was lower at higher altitudes this would suggest lower seed germination and survival at higher altitudes. We predicted that *H. canterburyana* would have asynchronous reproduction across elevations due to flowering commencement triggered by temperature [14,73]. Our studies indicated that reproductive output and timing was variable for plants both within and among sites at different altitudes, which has similarly been reported across the environmental range of a Seychelles palm [41]. Asynchrony in flowering across altitudes is hypothesised as a potential barrier to gene flow, resulting in genetic structuring or clustering along altitude gradients [43,44,74]. 

### 2.4. Population Genetics

Genetic diversity was relatively high (*A* 3.7, *P* 97.5%, *He* 0.433) but with little variation across the sites; 50 alleles were recorded across the ten loci tested (Table 4). There was no significant correlation (*p* > 0.05) between growth rate similarity and genetic similarity among individual plants (Table 2). However, there were significant negative correlations between aspect and Shannon’s diversity index (*I*; *r_s_* = −0.812; *p* < 0.05). There were no other significant correlations (*p* > 0.05) or differences in genetic diversity between the mountains, high and low altitudes, aspect or exposed and sheltered sites that could explain variation in the data. The genetic diversity in *H. canterburyana* is comparable, though moderately higher, than has been reported for endemic palms from the Seychelles, Vanuatu and Canary Islands [41,75]. *Hedyscepe canterburyana* has similar levels of genetic diversity compared with the other Lord Howe Island endemic palms [5,6,72] and also widespread Mexican palms that are considered to have higher levels of variation compared to other tropical plants [76,77].

**Table 4:** Summary of the mean genetic diversity measures across 10 loci for each *H. canterburyana* site on Lord Howe Island. *N*, number of samples; *A*, mean number of alleles per locus; *Ae*, mean number of effective alleles per locus; *P*, percentage of loci polymorphic; *I*, Shannon’s information Index; *Ho*, mean observed heterozygosity; *He*, mean expected heterozygosity; *Ap*, number of private alleles; *F*, mean allelic fixation index.

*Site*	*N*	*A*	*Ae*	*P (%)*	*I*	*Ho*	*He*	*Ap*	*F*
*HC1*	31	3.70 (0.616)	2.22 (0.368)	100	0.814 (0.173)	0.335 (0.099)	0.435 (0.083)	0	0.256 (0.174)
*HC2*	28	4.00 (0.632)	2.56 (0.541)	100	0.906 (0.179)	0.310 (0.092)	0.471 (0.081)	2	0.348 (0.176)
*HC3*	30	3.80 (0.663)	2.25 (0.457)	100	0.829 (0.155)	0.259 (0.067)	0.443 (0.067)	2	0.389 (0.150)
*HC4*	30	3.50 (0.671)	2.30 (0.485)	90	0.789 (0.194)	0.283 (0.076)	0.412 (0.091)	0	0.347 (0.160)
*HC5*	30	3.60 (0.670)	2.25 (0.410)	90	0.819 (0.178)	0.269 (0.090)	0.433 (0.083)	0	0.393 (0.183)
*HC6*	29	3.70 (0.496)	2.01 (0.365)	100	0.724 (0.168)	0.244 (0.070)	0.371 (0.084)	2	0.327 (0.127)
*HC7*	29	3.60 (0.379)	2.27 (0.434)	100	0.809 (0.173)	0.280 (0.092)	0.435 (0.081)	1	0.318 (0.165)
*HC8*	30	4.00 (0.650)	2.18 (0.307)	100	0.874 (0.158)	0.274 (0.089)	0.461 (0.068)	1	0.462 (0.185)
**									
*Total/Mean*	29.52	3.74	2.26	97.5	0.820	0.282	0.433	8	0.355

**Figure 5:**
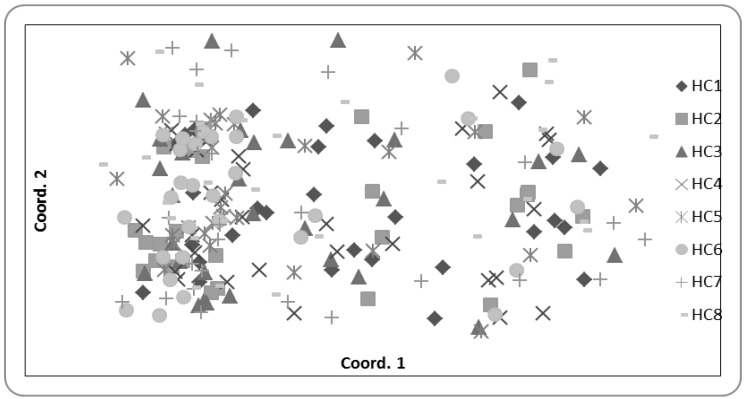
Plot of the Principle Coordinates analysis output showing the genetic relationship as measured at 10 microsatellite loci between all 237 individuals sampled across all populations, where symbols (see legend) indicate the population of each individual.

There was also very little evidence of genetic differentiation across the island distribution, with low *F_ST_* (0.030) and only 2% of the species genetic diversity distributed among sites, (Table 5). Consistent with high gene flow estimates (*Nm* 8.4; Table 5), 80% of the samples were assigned to a different site than their true location in the population assignment test indicating weak genetic provenance. The Principle Coordinates Analysis also demonstrated no clear genetic clustering of individuals associated with sites (Figure 5). Thus, the species appears to be acting as one panmictic population or as a metapopulation of connected subpopulations.

Low genetic differentiation reported here is similar to palms sampled across small spatial distances in Mexico and Brunei [76,78]. The other LHI palms have similarly low levels of genetic differentiation [6,72]. *Hedyscepe* *canterburyana* is abundant on LHI with good connectivity throughout its distribution. This suggests dispersal of either pollen or seeds over the entire distribution on the island. Lord Howe Pied Currawongs are expected to be an important seed disperser for this species [24]. Navascues *et al.* [79] hypothesised that stepping-stone gene flow along a continuous elevation gradient could counter balance the gene flow restrictions of asynchronous flowering. Such a mechanism may explain our results.

**Table 5:** Summary of species level diversity and partitioning of genetic variation across ten microsatellite loci and among eight *H. canterburyana* sites. Wrights F statistics (*F_IS_*, *F_IT_*, *F_ST_*), past gene flow among population (*Nm*), the mean number of alleles per locus within the species (*As*). In addition the results of an ANOVA analysis are given where the % of variability within and between population is given and the test statistic PhiPT significance values *** *p* < 0.001.

Statistic		Value	S.E
*F_IS_*		0.348	0.159
*F_IT_*		0.366	0.155
*F_ST_*		0.03	0.002
*Nm*		8.446	0.64
*As*		3.738	
Variation among populations			2%
Variation within populations			98%
PhiPT			0.015 ***

Despite evidence of gene flow across the island, all sites were moderately inbred (*F* 0.355; Table 4). At each site, six loci on average deviated significantly from Hardy-Weinberg expectations (*H-W*; *p* < 0.05). Sites with a north-west aspect (HC1, HC4) were more inbred (*F*; *r_s_* = −0.913) suggesting wind may play a role. This species is thought to be pollinated by insects with restricted ,movements which may lead to the formation of family clusters resulting in effective inbreeding [80]. However, there was no significant correlation (*p* > 0.05) between geographic and genetic distances within any site and very little evidence for spatial clustering of like genotypes within sites, suggesting little evidence of family genetic structuring. Thus, alternative scenarios provide more plausible explanations for inbreeding in *H. canterburyana*. Inbred yet genetically diverse populations of Belize palm *Chamaedorea ernesti-augusti* were explained by thrip pollinators movement among different groups of inflorescences in different years [81]. Inbreeding may also be due to a mixed mating system, pollination between close relatives or self-pollination between male and female flowers on the same plant [72,82]. Self-compatibility may enable a selection advantage whereby the frequency of genotypes that are successful under strong climate change selection can be increased [83]. We found that higher levels of inbreeding in *H. canterburyana* were correlated with reduced genetic diversity.

### 2.5. Population Growth Models

**Figure 6:**
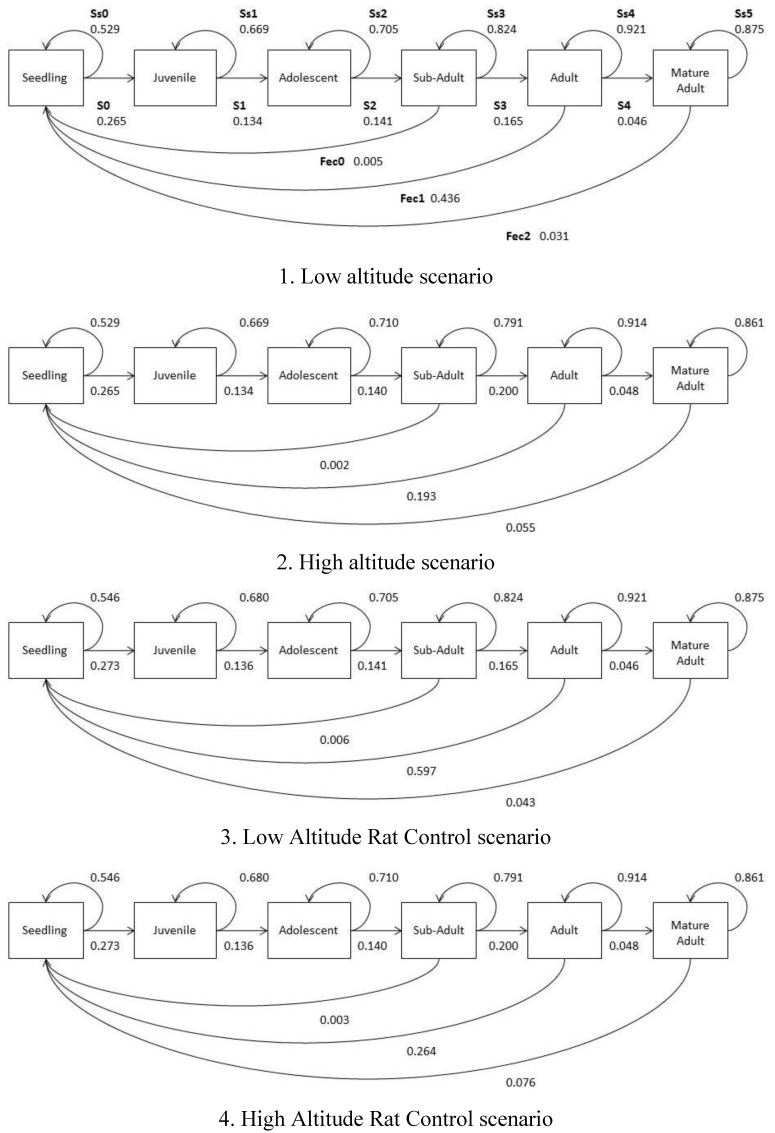
The lifecycle diagram of *H. canterburyana* on Lord Howe Island, and values previously calculated for matrix table. *Ssn*, stage-specific survival rate; *Sn*, transition survival rate; and *Fecn*, fecundity.

Population dynamic models and observational studies are extremely useful for quantifying recent climate induced upslope range shifts [19,22,84]. There were some limitations to the population model used in this study, including intrinsic restrictions by using static population data and the partially unequal distribution of individuals within stage classes [85]. Overall carrying capacity (*K*) and the initial stage abundance and residence estimations differed between “low altitude” and “rat management” models, and the “high altitude” models based on the input data (Table 6; Figure 6). Transition between stages (*S*) and survival likelihood varied depending on development stage and the model, reflecting the population structure and grow rate characteristics (Figure 6). The “high altitude” model had the greatest “Mature Adult” *Fec*, reflecting the observed greater reproductive output, at high altitudes (Figure 6). Thus, we believe the models provide a reasonable representation of population dynamics for *H. canterburyana*, given the evidence of low recruitment in comparison to other palms, and are therefore appropriate for use in evaluating population growth differences at different altitude [30,86].

**Figure 7:**
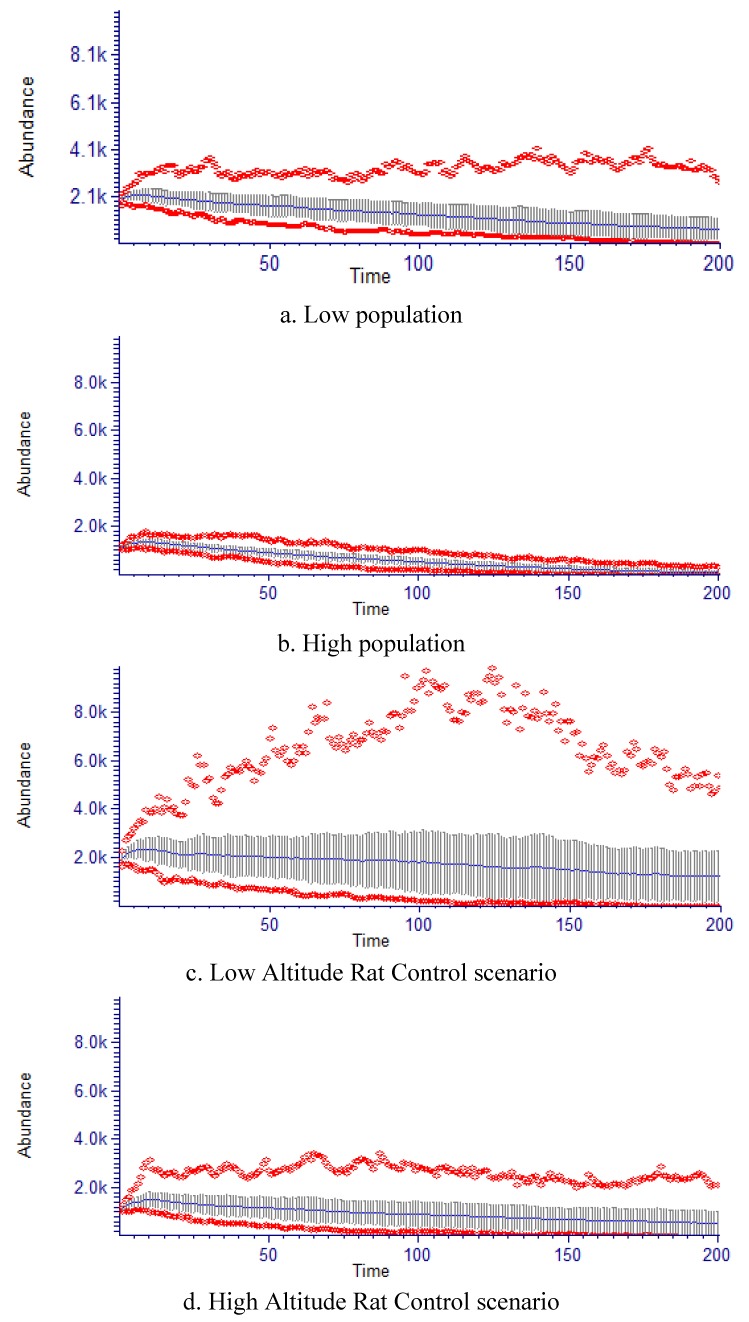
RAMAS probability trajectory summaries for low altitude, high altitude and associated rat control scenarios of *H. canterburyana* for 200 years. Line is mean value with 1 vertical standard deviation bars. Red diamonds are minimum and maximums. Models were run for populations in one Hectare.

Our model matrices predicted slow population declines for *H. canterburyana* with the “high altitude” model indicating considerably greater declines in numbers compared to the low altitude model (Table 6). We predict that the high altitude populations will start to significantly decline after approximately 30 years and could be reduced to as low as 8% of the current population size after 200 years, with a 69% chance that they will become locally extinct from the high altitude part of their distribution within 180 years if there is no effective reduction in rat predation (Table 6; Figure 7). In contrast to expectations, the models predict a slower population decline at lower altitudes, with these sites potentially retaining 55% of the initial population size after 200 years . Low altitude sites had and only a 3% risk of extinction at lower altitudes within 200 years (Table 6; Figure 7). While upward expansion of plant taxa may occur at different rates, altering community composition [18], Crimmins *et al*. [42] suggest that range could also shift, expand or contract to low altitudes, evidenced by increased population growth at the lower end of a species elevation range. This is more consistent with our results. *Hedyscepe canterburyana* has an overlapping distribution with *Howea belmoreana* at the lower end of its elevation range, thus there is potential for increased competition between these species.

**Table 6:** Population model outcomes for *H. cantaburyana* on Lord Howe Island at different altitudes and with rat control. The carrying capacity per hectare *K* (Ha), growth rate (λ, approximate finite rate of increase),starting population size per Hectare, mean final population size (n+t) for each model, predicted percentage of initial population remaining after 200 years, probability and time to quasi-extinction (population falling below 100 individuals) with probability. For mean predicted values the range ±1 one standard deviation or the 95% confidence interval are given in brackets.

Model	*Low altitude*	*High altitude*	*Low altitude rat control*	*High altitude rat control*
Starting population (/Ha)	1,886	1,109	1,886	1,109
Final population (n + t) (/Ha)	652 (195–1,109)	85 (0–171)	1,246 (234–2,258)	560 (78–1,042)
*% remaining @200 years*	55%	8%	66%	50%
*K (Ha)*	2,220	3,770	2,220	3,770
*λ*	0.9968	0.9721	1.0108	0.9835
Quasi extinction probability	0.030 (0.000–0.119)	0.690 (0.601–0.779)	0.020 (0.000–0.109)	0.080 (0.000–0.168)
Time to Quasi extinction	>200 years (*p* = 0.01)	181.1 years (*p* = 0.02)	>200 years (*p* = 0.01)	>200 years (*p* = 0.01)

Rat control measures are currently undertaken on eight % of LHI [54]. Seedling survival value (*S* + *Ss*) and fecundity values were greater for the “rat management” simulations, consistent with Auld *et al*. [24]. Our models predict that rat management has considerable positive impacts for the species population survival at both altitudes (Table 6). The most striking impacts were in the “high altitude rat management” simulations where the probability of quasi extinction was reduced to only 8% (from 69% where there is no rat management) and population decline slowed to 50% (as opposed to 92% for no rat management) (Table 6; Figure 7). Negative growth rates have also been found for South American palm *Geonoma schottiana* due to herbivore predation [87]. Significantly increased seedling recruitment of *Rhopalos tylis sapida* after Pacific rats were eradicated from several small islands, revealed a previous underestimation of rat predation on that species [88]. The moderate reduction in population decline in these models following real-life rat management actions on islands is sufficient to boost population growth and recovery [88,89].

### 2.6. Climate Change and *In Situ* Persistence

Phenotypic plasticity may buffer individuals against the short term effects of climate change [32,34,38,39]. Growth rate of some palms species varies between individuals in a plastic response to environmental conditions [41,90]. Variation in leaf scar interval occurred within *H. canterburyana* sites across its altitude range but was not correlated with genetic similarity (*p* > 0.05) and hence, growth rate variation seems more likely a plastic response to the local environment.

Climate linked genetic variation in natural populations may increase the likelihood that some individuals may be pre-adapted to future climatic conditions [35,36,37]. Isolated island endemics that have survived through historical climatic changes potentially have climatically adapted genotypes [91]. *Hedyscepe canterburyana* or an ancestor has persisted on LHI for at least 4 million years, through several periods of climatic change [5,92]. The moderate to high levels of genetic variation and diversity for *H. canterburyana* were well dispersed across elevation, therefore individuals potentially exist within current populations that are genetically pre-adapted to a varying climate [93].

## 3. Experimental Section

### 3.1. Field Methods

Samples of *H. canterburyana* for genetic analysis were collected in 2007 from eight sites across the species distribution on Lord Howe Island (Figure 1). There were four sites per mountain (Mt Lidgbird and Mt Gower) with four sites at both high altitudes (776–875 m) and low altitudes (428–479 m; Figure 1). Geographic location was recorded for each site using a Garmin Etrex GPS. Thirty plants were sampled from each site (total 240). Within each site, plants were randomly sampled from all size classes present, with every second or third plant encountered being sampled. Fresh leaf material was collected and dried in silica gel. The relative locations of the individuals sampled within each site were determined by measuring the compass bearing and distance between plants and later converting these to X and Y coordinates.

In November 2008 several temperature I-buttons (Thermodata Pty Ltd.) were installed along an altitude gradient of 10–800 m in the Erskine Valley to the summit of Mt Gower. Temperature was recorded every three hours for a nine-month period to July 2009, however, reliable data were only received for the first eight months for I-buttons at eight locations.

In 2008, permanent plots were established to monitor the *Hedyscepe canterburyana*–*Howea belmoreana* intergrade at three locations (Figure 1). At each site, three transects of 5 m wide × 40 m long were run perpendicular to the slope at different elevations representing: (i) upper dominated by *H. canterburyana*; (ii) lower dominated by *H. belmoreana*; and (iii) intermediate both species. Within each transect, locations of each individual of the two study species was noted and its height (cm) recorded [70]. Circumference of the trunk at 50 cm height, number of inflorescences and stage (new, old or fruiting) number of leaf scars for individuals with a trunk or the number of leaf scars in a two metre section was recorded as per Auld *et al*. [24]. Many palms are characterised by easily counted leaf scars, which can be used to estimate growth rate and plant age [24,29,64].

To measure variation in demographic structure sixteen sites were sampled in 2010 over the full elevation range (316–850 m), with seven sites on Mt Lidgbird and nine sites on Mt Gower (Figure 1) outside of rat control areas. Six sites were at the approximate location of genetic sampling in 2007 (HC1-6). Six sites utilised the long term monitoring plots (HC9-14; Figure 1). 

Each of the 16 sites were systematically surveyed in 10 m wide belt transects perpendicular to the slope. Data for all *H. canterburyana* individuals were recorded until 30 individuals with an emergent trunk were sampled from the site. Locations of all individual plants were mapped as XY coordinates (m) relative to the start point using a Laser Distance meter (Leica DISTO D5, Leica Geosystems) as this provides more accurate relative location than a hand held GPS under canopy cover and on steep slopes. For each *H. canterburyana,* the height of individuals with no emergent trunk was measured to the tip of the tallest leaf, for plants with a trunk, the presence of a trunk was recorded and height measured to the base of the leaf sheath using the Laser Distance meter. The number of leaf sheath scars (rings) on the trunk were counted as per Auld *et al*. [24], and diameter at breast height (*DBH*) was measured using a diameter tape. The number of reproductive structures were recorded in categories: newly developing, half sized green fruit, full size green fruit, full sized mottled fruit, full sized red fruit or aborted. The abundance of fruit on each infructescence was estimated in abundance classes of: less than 10 fruits, 11–20 fruits, 21–50 fruits, 51–100 fruits.

The following data were recorded at each site: altitude (*Alt*), cardinal direction of the aspect of the slope (*Asp*), and transect using a compass (Suunto Co., Finland), slope of land (degrees) using a hand held manual inclinometer (Suunto Co.) and the location at the start and end of the transect using a Garmin Etrex GPS. Orographic cloud cover often forms at altitudes above 550 m [14]; thus sites greater than 550 m were designated as high populations experiencing more frequent orographic cloud cover (*H*), the remainder designated as low populations (*L*). Whilst undertaking fieldwork, it was observed that there is considerable shade cast on some Erskine Valley sites by Mt Lidgbird to the north.Sites in the Erskine Valley were therefore designated as protected from sun exposure in this manner (*Pro*) or exposed (*Ex*).

### 3.2. Genetic Analysis

Leaf tissue was frozen in liquid nitrogen then ground and DNA was extracted using a DNeasy Plant Mini-kit (QIAGEN) [74]. Thirty-six published microsatellite primers developed for palm species were tested for cross-amplification in *H. canterburyana* [94,95,96,97]. DNA samples from the original species for which they were developed (positive control) and a blank sample (negative control) were used to enable identification of artefacts. Only loci that amplified in the original test species size range were considered.

Ten microsatellite primer pairs were used for *H. canterburyana* in this study: from *Phoenix dactylifera* by Billotte *et al*. [94] mPdCIR25 (Pd25), mPdCIR050 (Pd50), mPdCIR032 (Pd32), mPdCIR090 (Pd90); *Euterpe edulis* by Gaiotto *et al*. [96] mEe15 (Ee15), mEe03 (Ee3), mEe54 (Ee54); *Elaeis guineensis* by Billotte *et al*. [95] mEgCIR0476 (Eg476), mEgCIR0391 (Eg391); and *Oenocarpus bataua* (Ob7) [97]. The forward primer was end-labelled with a fluorescent dye (HEX, FAM or NED), except for Ob7, which was end-labelled with HEX following the universal florescent labelling method of Shimizu *et al.* [98].

PCR was performed using reaction volumes of 12.5 µL containing, 1× PCR buffer (Fisher Biotech), 200 µM of each dNTP, 1.5 mM MgCl_2_ (Fisher Biotech), 0.2 µM forward primer, 0.2 µM reverse primer and 0.5 U F1 *Taq* polymerase (Fisher Biotech), optimised for individual loci as follows: Eg476 2 mM MgCl_2_; Pd90 125 mM dNTP, 2 mM MgCl_2_; Ob7 125 mM of dNTP, 2.5 mM MgCl_2_, 0.4 µM forward primer, 0.1 µM reverse primer and 0.1 µM HEX; Ee03 0.52 µM forward primer and reverse primer; Ee54 and Ee15 250 µM dNTP.

The PCR cycling conditions for Pd25, Pd50, Pd32, Eg391, Eg476 and Pd90 were): 95 °C for 1 min; 35 cycles of 94 °C for 30 s, 52 °C for 1 min, 72 °C for 1 min; 72 °C for 8 min [94,95]. Optimised of cycling conditions for individual loci as follows: annealing temperature for: Pd50 and Eg391 49 °C; Eg476 56 °C; Pd90 48.6 °C. The PCR conditions for Ee03, Ee15 and Ee54 followed Gaitto *et al*. [96]: 96 °C for 2 min; 30 cycles of 94 °C for 1 min, 59 °C for 1 min, 72 °C for 1 min, 72 °C for 7 min. For Ee54, annealing temperature was 56 °C. The PCR conditions for Ob7 followed Montufar *et al*. [97]: 96 °C for 2 min; 35 cycles of 95 °C for 30 s, 55 °C for 30 s, 72 °C for 45 s, 72 °C for 10 min.

PCR products of Pd25, Eg 476, Ob7 and Ee3 were run on a Gelscan 2000 (Corbett Research) fragment analyser, using the CR Gelscan program v 7.2.7 (Corbett Research 2001) to visualise the results. The scored bands were assigned to alleles using the oneD Scan v2.05 program (Scanalytics Inc). The PCR products from the remaining six loci were run on a AB3500 Genetic Analyser (Applied Biosystematics 2010). Fragments were scored into alleles at each locus using the ABI Gene Mapper v.4.1 program (Applied Biosystematics 2010) and cross-checked manually. 

### 3.3. Statistical Analysis

The allelic frequencies, and measures of genetic diversity, mean number alleles per locus (*A*), the effective number of alleles (*Ae*), the number of private alleles (*Ap*), percentage polymorphic loci (*P*), mean expected heterozygosity (*He*), mean observed heterozygosity, (*Ho*), the information index (*I*) and the inbreeding coefficient (*F*) were calculated. A chi-square analysis was undertaken at each loci to determine significant deviation from Hardy-Weinberg equilibrium in GenAlEx V6.4 [99].

Spatial analysis was undertaken to determine the density (*D*; plants/Ha) of *H. canterburyana* at each site; average distance to nearest neighbour (*NND1*) and three nearest neighbours (*NND3*) and pairwise geographic distance within sites between each individual sampled for genetics was calculated using GenAlEx V.6. Carrying capacity (*K*) per Ha for the high and low altitude population growth models was estimated by doubling the mean density (*D*) for high altitude sites (HC5, 6, 15–18) and low altitude sites (HC1-4, 9–14), based on the assumption that *H. canterburyana* was not occupying the full potential carrying capacity and that twice the current density was a conservative yet realistic estimate of the increase sites could accommodate. Number of leaf scars (*H_2_*) for trunked individuals was extrapolated using the mean number of rings in 1 m multiplied by mean trunk height (*H_1_*). Average growth rate (number of rings per metre; *R/m*) for each population was calculated as: *H_2_*/*H_1_.* Growth progression rate (*rR/m*) for high sites relative to low sites the formula (*R/ml)*/(*R/mh*) was used, where *R/mh* was mean growth at high and *R/ml* low altitudes sites*.*

Individuals were assigned to fifteen size classes; three size classes for non-trunked individuals using height increments of 0.6 m and crown size increments of 0.6 m and twelve size classes for trunked individuals using trunk height increments of 0.5 m. The proportion of individuals in height classes for each site and across the whole distribution was plotted. The number of inflorescences was plotted against height classes and the correlation between number of multiple inflorescences on each reproductive individual and *DBH*, *H_1_* and *H_2_* was tested using Spearman’s correlations. The strongest significant correlation *H_1_* was determined to be the best indicator of reproductive age.

Developmental stages for later use in the population growth models were calculated based on the number of inflorescences for each height class, the approximate height of reproduction commencement and peaks in reproduction [68]. Thus, stages were defined as: Seedlings (*S*; non-reproductive plants <60 cm), Juveniles (*J*; non-reproductive plants >60 cm), Adolescents (*Ad*; non-reproductive individuals trunk <0.5 m), Sub Adults (*SA*; trunk height 0.5–1.5 m, ≤2 inflorescences), Adults (*A*; trunk height 1.5–5 m, >2 inflorescences) and Mature Adults (*MA*; trunk >5 m).

The mean number of inflorescences per reproductive individual (*Inf*) was calculated for each site and overall (*Infp*). The percentage of reproductive individuals per site (% *Inf*), the proportion of individuals of reproductive age (*RI*) with reproductive structures present per site, and the effective population size based on reproduction [100] were calcualted. The mean number of seedlings per *RI* (*nS/RI*) was calculated for each site and for entire population. Fecundity values (*Fec*) for the population growth models were derived from the mean *nS/RI* for high (*nS/RIh*) and low (*nS/RIl)* sites. The proportion of inflorescences at each stage of maturity was calculated for each site. Thus, maximum reproductive output (*MRO*) for each *RI* was calculated as the sum of the maximum value for each abundance class of all inflorescences. In addition, *MRO* for immature (*iMRO*) and mature sized (*mMRO*) fruit was calculated to enable testing for evidence of potential rat predation. 

Significant differences in demographic and genetic measures among sites and among *Asp* were tested using Analysis of Variance (ANOVA) in SPSS v.19 (IBM 2010). Differences in demographic, growth (*R/m* and *DBH)* reproductive (*MRO* and *mMRO*) and genetic measures between mountains, *H* and *L*, and *Ex* and *Pro* sites were tested for significance using ANOVA or Independent T-tests. Spearman’s rank correlations were used to test for significant relationships of *Alt* and *Asp* with demographic, growth rates (*R/m*, *DBH*), reproductive (*MRO* and *mMRO*) and genetic measures in SPSS v.19 (IBM 2010).

Measures of partitioning of genetic diversity in the species (*F_ST_*; *F_IS_*; *F_IT_*), were calculated using Wright’s F statistics [101] and measures of gene flow (*Nm*) were calculated based on *F_ST_*. The significance of the genetic partitioning among populations within the species was statistically tested by AMOVA (9999 permutations). Genetic effective population size (*Ne*) for the species was calculated, using the GenAlEx v.6.4.

Nei’s genetic distance was calculated between individuals and the sites and Principal Coordinates Analysis (PCoA) was used to investigate the major patterns in genetic relationships among all individuals sampled, using 9999 bootstrap permutations GenAlEx V6.4. The genetic distinctiveness of sites was tested using a frequency-based population assignment test undertaken using 9999 bootstrap permutations. A Mantel test between genetic distance (Nei) and geographic distance using and a spatial autocorrelation analysis were used to test for spatial genetic structure within sites, using 9999 permutations using GenAlEx V6.4. Within sites, spatial autocorrelation analysis was undertaken using three annulus distance classes (5 m, 10 m, 15 m), based on general average seed dispersal [102].

Mean daily minimum and maximum temperature was calculated for each altitude. Differences between altitudes were tested using ANOVA, and the strength of the relationship with altitude was tested with Spearman’s Rank correlations in SPSS v.19. Where correlations were significant, they were regressed to estimate adiabatic decline in temperature.

Long term historical climatic data for Lord Howe Island were obtained from the Australian Bureau of Meteorology (BOM). Climatic data for 1960 to 1987 were obtained from the original Lord Howe Island Meteorology Station (200440); and for 1988–2010, from the Lord Howe Island Aero Meteorology Station (200839). Only years with a BOM “100% completeness” rating were utilised. To ensure that weather records between the two weather stations were not affected by microclimatic variation, records for the daily, mean, maximum and minimum temperatures and rainfall at the two stations were compared in the month of overlap (November 1988). While some differences were noted in the records, they were not greater than ±0.01 °C for temperatures or ±1 mm for rainfall, and therefore were not regarded to contribute to any error in the statistical analysis of long term weather records for LHI.

To estimate the climatic changes on LHI 1940–2010, mean daily temperatures (minimum and maximum) were calculated for each month and year, and total annual rainfall was calculated for each year. Decadal differences in mean annual and monthly minimum and maximum temperatures, and mean annual rainfall, were tested with ANOVA. The strength of climatic changes over time were tested using a Spearman’s rank correlation and regressed against decade.

A Euclidian resemblance (similarity) matrix was created based on the environmental attributes (Alt, Asp, Mountain, mountain shade) of the sites and two Bray-Curtis similarity matrices were created using *Hedyscepe* population structure attributes (1NND, 3NND, development stage proportions), and inflorescence attributes (*RI*, *Ip*, *MRO*) in PRIMER v.6.1.5 (PRIMER-ELtd 2006). For each resemblance matrix, multi-dimensional scaling analysis was undertaken. The strength of the relationship between the Euclidian resemblance matrix and each Bray-Curtis similarity matrix was tested with a rank correlation in randomly permuted samples (999 permutations) using the RELATE analysis (equivalent to a Mantels test) in PRIMER v.6.1.5 (PRIMER-ELtd 2006). In addition, Euclidian distance was used to calculate a distance measure between individual trunked plants growth rates based on the number of leaf scars per metre. This was then tested for correlation with genetic distance among the same plants as above using PRIMER v.6.1.5.

### 3.4. Population Growth Models

Four stage matrix models with a time step of one year were constructed for *H. canturburyana* (Figure 5) [103]. These were: a “*low altitude*” model based on static population parameters at low altitudes; a “*high altitude*” model based on static population parameters at high altitudes, and “*rat management*” models for high and low altitudes with increased fecundity and seedling survival to simulate the removal of rats. Initial stage abundances were calculated for a one hectare (Ha) population sample based on the mean proportion of individuals in each stage and the mean number of individuals per hectare across “*L*” sites for “low altitude” and “rat management” models and “*H*” sites for the “high altitude” model. 

Stage transition rates were estimated using paired height class data from repeated surveys in established permanent plots. The average time spent in each class (*Ayhc*) was estimated based on differences in the proportion of the individuals in each class over a three-year timeframe. This was converted to number of years of residence in each stage (*y*). A frequency table was constructed to estimate for each stage, the transition probability values for survival, the probability of moving to the next stage (*pSn*) in one year (one time step), and the probability of stage specific survival (*pSsn*) in one year. These values were used for “low population” model. The average time spent in each height class for the “high population” model was calculated using *rR/m* × *Ayhc*, and a frequency table of transition probability values was constructed for “high population”.

The combined static height class structure (2010) was utilised to calculate the overall survivorship from one stage to the next stage (*NS*), the using the formula *NS* = (N(*t* + 1)/N(*t*))^1/*y*^ which accounts for mortality. Transition survival rate (*Sn*) was estimated using the formula: *Sn* = *NS* × *pSn*. Stage specific survival (*Ssn*) was estimated using the formula: *Sn* = *NS* × *pSsn*.

Relative reproductive output (*RRO*) was calculated for each reproductive development stage using the total *MRO*. Fecundity (*Fec*) for each stage was calculated using the formula: *Fecn* = *RROn* × *nS/RI_n_*. Fecundity values for the “*rat management*” model were calculated using the *Fec* values as above ×37% for both high and low altitude, as this is the median for observed fruit loss [24]. Survivorship from “Seedling” to “Juvenile” stage for the “rat control” simulations was increased by 10%, based on Auld *et al*. [24]. This was then incorporated into the stage matrix section of the model. Average stage residencies were examined as a result a second adult stage was incorporated into each model (“Adult 2”) with the same stage matrix values as the “Adult” stage, to best reflect relative residency times for each stage in the population modelling algorithms.

RAMAS GIS metapopulation v.5.0 software (Applied Biomathematics 2005) was used to develop stage-structured stochastic models of the potential population growth of the “*low altitude*”, “*high altitude*”, and “*rat management*” populations. Matrix models with seven stages were based on the stage matrices table [103]. The standard deviation of the matrix and of K was set at ±10%, to allow for measurement errors [104]. Density dependence in population dynamics was modelled with scramble type (logistic, Ricker) to effect all vital rates, weighted for seedlings = 0.0001, juveniles = 0.001, adolescent = 0.25, sub-adults, adults and mature adults = 1. Within the model framework, demographic stochasticity was selected and environmental stochasticity was designated ‘lognormal’. Dispersal was set to zero. Maximum growth rate (*Rmax*) was set to a neutral value of 1 and quasi-extinction thresholds were set at 100 individuals. Each population model projected the abundance over 200 time steps (200 years), outputs of the mean of 100 replications. Predicted population growth (lambda λ), differences between starting population size and mean final population size, and time to quasi extinction, were compared between models.

## 4. Conclusions

We found that Lord Howe Island is getting warmer and drier and quantified the degree of temperature change with altitude (0.9 °C per 100 m), which is greater than rates often used to make climate change predictions. For *H. canterburyana*, differences in development rates, population structure, reproductive output and population growth rate were identified between altitudes. In contrast to expectations, the highest recruitment was at low altitudes and we found no evidence of upward range contraction of *Hedyscepe canterburyana* in response to recent climate change. We found that trunked *H. canterburyana* individuals had smaller diameter at breast height and were shorter at higher altitudes. Conversely, reproductive output was higher at higher altitudes under cloud cover. *Hedyscepe canterburyana* has relatively high genetic diversity distributed across the island, increasing its effective population size and its potential for persistence through environmental change. Genetic composition was not correlated with individual growth rate variation, suggesting the species has a plastic growth response. The presence of both genetic diversity and phenotypic plasticity indicates some potential for both a short-term buffer to climatic variability and the potential for genetic adaptation to occur in the long term.

High and low altitude populations of the species are predicted to slowly decline under ongoing rat predation with high altitude sites affected the most. We predict that the high altitude populations under current conditions will start to decline significantly in approximately 30 years, and there is a 70% chance of extinction within 180 years. However, the low altitude populations are not predicted to become extinct within the next two hundred years. We found that rat control would lead to a significant reduction in population decline particularly at high altitudes, and is likely to be more important for survival of this species than climate change. Thus, long term management aimed at rat control will be the most effective action to benefit this species. While rat eradication would be the most effective action, an alternative of rotating rat control measures around the island over time should enable cohorts of seedlings to establish in different environments.
